# In search of elixir: Pharmacological agents against stem cell senescence

**DOI:** 10.22038/ijbms.2021.51917.11773

**Published:** 2021-07

**Authors:** Hourieh Tousian, Bibi Marjan Razavi, Hossein Hosseinzadeh

**Affiliations:** 1 Vice-chancellery of Food and Drug,Shahroud University of Medical Sciences, Shahroud, Iran; 2 Targeted Drug Delivery Research Center, Pharmaceutical Technology Institute, Mashhad University of Medical Sciences, Mashhad, Iran; 3 Department of Pharmacodynamics and Toxicology, School of Pharmacy, Mashhad University of Medical Sciences, Mashhad, Iran; 4 Pharmaceutical Research Center, Pharmaceutical Technology Institute, Mashhad University of Medical Sciences, Mashhad, Iran

**Keywords:** Cellular senescence, Endothelial progenitor cells, Induced pluripotent stem-cells, Melatonin, Mesenchymal stem cells, Telomerase

## Abstract

Stem cell senescence causes different complications. In addition to the aging phenomenon, stem cell senescence has been investigated in various concepts such as cancer, adverse drug effects, and as a limiting factor in cell therapy. This manuscript examines protective medicines and supplements which are capable of hindering stem cell senescence. We searched the databases such as EMBASE, PubMed, and Web of Science with the keywords “stem cell,” “progenitor cell,” “satellite,” “senescence” and excluded the keywords “cancer,” “tumor,” “malignancy” and “carcinoma” until June 2020. Among these results, we chose 47 relevant studies. Our investigation indicates that most of these studies examined endothelial progenitor cells, hematopoietic stem cells, mesenchymal stem cells, adipose-derived stem cells, and a few others were about less-discussed types of stem cells such as cardiac stem cells, myeloblasts, and induced pluripotent stem cells. From another aspect, 17β-Estradiol, melatonin, metformin, rapamycin, coenzyme Q10, N-acetyl cysteine, and vitamin C were the most studied agents, while the main protective mechanism was through telomerase activity enhancement or oxidative damage ablation.

Although many of these studies are *in vitro*, they are still worthwhile. Stem cell senescence in the *in vitro* expansion stage is an essential concern in clinical procedures of cell therapy. Moreover, *in vitro* studies are the first step for further *in vivo* and clinical studies. It is noteworthy to mention the fact that these protective agents have been used in the clinical setting for various purposes for a long time. Given that, we only need to examine their systemic anti-senescence effects and effective dosages.

## Introduction

Adult stem cells distributed throughout the entire body are responsible for tissue regeneration. However, human beings are not immortal and experience the aging phenomenon, as well as age-related diseases (1). Aging has received much attention in recent years, while stem cells play a crucial role in aging. It is established that the lack of sufficient stem cell regeneration leads to the aging phenomenon. Telomere shortening, DNA damage, harmful epigenetic modification, and depletion of proliferative potential all induce permanent cell cycle arrest, called cellular senescence. Senescent stem cells lose their functions for tissue rejuvenation and perseveration (2). Their high regeneration capacity or serial transplantation increases the rate of DNA damage and leads to telomere erosion, reactive oxygen species (ROS), and ultraviolet (UV) mutagenicity. Studies have demonstrated the accumulation of DNA damage in the stem cell aging process. This damage could affect cellular function or transform cells into cancerous types. Telomeres contain thousands of base pairs of repetitive DNA sequences, TTAGGG repeats, which protect chromosomes from end-to-end fusions. Each round of cell division reduces telomere length unless the cell expresses telomerase. Various checkpoints and mechanisms in the cell cycle monitor DNA integrity and fix most of the mutations. If during this monitoring the genome repair systems fail, apoptotic signals will clear any damaged cells. However, if the involved protein cascades are not strong enough to trigger apoptosis, these cells will permanently stop in the cell cycle; and this state is called cellular senescence. Senescence is a protective barrier against cancerous cell formation (3, 4). P53, p16, p19 (p14 in humans), and retinoblastoma protein (Rb) are tumor suppressor proteins playing a role in the cell cycle checkpoints. They negatively control cell division to reinforce DNA repair, and while genomic damages accumulate by age, their quantity increase in the cell. P19 promotes p53 protein stability by inhibiting Mdm2-mediated p53 degradation (5). P21 and p16 are cyclin-dependent protein kinase (CDKs) inhibitors. CDKs phosphorylate Rb and inactivate it to promote the cell cycle (6). A low concentration of p21 activates CDKs 4 and 6 and promotes the cell cycle; however, a high concentration of p21 suppresses cyclin E-dependent kinase 2 and arrests the cell cycle (7).

Senescent cells secrete pro-inflammatory factors such as IL-2, IL-6, IL-8, and TNF-α to attract the immune system to clear them. The high number of senescent stem cells as a result of the high rate of formation or low rate of clearance puts the body in a chronic inflammatory state. This condition causes more senescent cells, aging, and various non-aging pathologies such as metabolic syndrome or adverse drug effects (8).

There are different approaches to prevent stem cell senescence, such as using hypoxic conditions (9), exosomes or extracellular vesicles secreted from stem cells (10), supportive matrices or cells (11, 12), microRNAs regulation (13), and gene therapy (14). In addition to these approaches, we can use pharmacotherapy. Pharmacotherapy is a convenient and economical tool that can be applied for systemic aims. For this kind of intervention, there are various natural substances derived from plants and non-natural agents. We have reviewed natural agents previously (15). Therefore, this paper has outlined some problems related to stem cell senescence (16) and all studies about protective medicines and supplements also their protective mechanisms (Tables 1 and 2).

## Materials and Methods

We searched databases such as Embase, PubMed, and Web of science with the keywords “stem cells,” “progenitor,” “satellite,” and “senescence.” On account of stem cell senescence being considered a therapeutic method in cancer therapy, we excluded the keywords “cancer,” “tumor,” “malignancy,” and “carcinoma.” The results were without a time limitation until 2020/06/01. We categorized the protective agents as natural (from plants) and non-natural. Following our review of protective natural agents in our previous paper (15), for the present paper, we selected 47 articles among 2137 results that investigated the protective effects of medicines and supplements which inhibit cellular senescence in various types of adult stem cells.

## Results

***Protective medicines inhibit adult stem cell senescence***

*Endothelial progenitor cells (EPCs)*

Angiogenic progenitor cells with three surface markers CD133, CD34, and VEGFR-2 are called endothelial progenitor cells, which predominantly locate in bone marrow (BM). When they migrate to the bloodstream and express endothelial markers such as VE-cadherin, eNOS, and von Willebrand factor, their phenotype gradually changes. These cells are involved in re-endothelialization and neovascularization. EPC sources are BM or peripheral blood. Various factors, such as drugs, diseases, and growth factors, influence their number and migratory activity (17). For example, atherosclerotic and obese patients have premature EPC senescence (18, 19). Senescent EPCs have shown a finite migratory and proliferative potential for cell therapy in ischemic diseases (20). Patients suffering from migraines have more senescent EPCs with less migratory capacity leading to the conclusion that there is a possible link between a higher risk of cardiovascular diseases and migraine (21). Angiotensin II (Ang II), which causes hypertension, can accelerate EPC senescence and reduce their differentiation capacity (22). Doxorubicin is an antineoplastic agent that significantly increased the risk of heart failure. This anticancer drug via Nox2 (an NADPH oxidase) could induce EPC senescence (23). Also, studies showed that the rate of senescent EPCs had increased in pre-eclampsia (24).

***In vitro*** **studies**

 According to a study, aspirin (a cyclooxygenase inhibitor that prevents platelet aggregation in cardiovascular disease, 50-100 mM) protected h-EPCs against senescence and improved their migratory and adhesion ability. The authors declared the protective mechanism was not via eNOS (25). 

Coenzyme Q10 (a vitamin-like substance that is also made naturally in the body, ten µM) phosphorylated AMPK, thus increased Akt/eNOS phosphorylation, HO-1 expression, which in turn reduced ROS and high glucose-induced senescence in EPCs of healthy volunteers. In this pathway, NO protected EPCs against high glucose (26). 

Statins are anti-hyperlipidemia medicines that inhibit HMG-CoA reductase. Atorvastatin (1 μM) increased Akt phosphorylation and telomerase activity, thus reversed homocysteine-accelerated senescence in h-EPCs (27). Atorvastatin (0.1 μM) and mevastatin (0.1 μM) decreased cellular senescence in h-EPCs. The protective mechanism was independent of NO, ROS, and telomerase activity. In this study, atorvastatin up-regulated cell cycle proteins such as cyclin A, cyclin F, and down-regulated p27 in a dose-dependent manner via the PI3K/Akt pathway. Maybe the inactivation of FOXO by Akt could result in p27 down-regulation (28). Atorvastatin at the concentration of 0.1 μM activated the Akt pathway without affecting telomerase activity. However, the higher concentration of atorvastatin (10 fold) increased the Akt phosphorylation and telomerase activity. Telomerase activation through Akt may be dose-dependent in the EPCs. 

 The physiological range of insulin (0. 1, 1 nM) increased Akt phosphorylation, and eNOS expression in rat bone marrow generated EPCs in high and normal glucose conditions (29). 

Angiotensin-2 (Ang2) decreased telomerase activity, increased peroxynitrite, superoxide, and AT1R mRNA and protein expressions in h-EPCs. Pre-treatment with pioglitazone (PPAR- γ agonist, an anti-diabetic drug, 10 μM) reversed these effects and decreased senescence in h-EPCs (30). Peroxynitrite and superoxide could damage the cellular genome. AT1R increases the action of Ang2 on cells. As mentioned before, PPARγ like Nrf2 controls the expression of HO-1at the transcriptional level. Then, HO-1 reduces the activity of NADPH oxidase and consequently the content of intracellular ROS (31).

Hormone replacement therapy with 17 beta-estradiol has positive effects on cardiovascular diseases in menopausal women (32). In a study, 17 beta-estradiol (100 nM) inhibited Ang2, reduced telomerase activity, increased AT_1_R mRNA and protein expressions, and elevated gp91phox (NOX2) mRNA expression, thus inhibited h-EPC senescence. Ang2 induces peroxynitrite formation. Subsequently, peroxynitrite activates redox-sensitive NF-κB and leads to AT1R up-regulation. This study also showed that 17 beta-estradiol inhibited the p38/MAPK pathway (33). MAPK and p38 activation contribute to p53-induced replicative senescence (34). In another study 17, beta-estradiol (100 nM) increased Akt phosphorylation and telomerase activity and led to lower EPC senescence. These EPCs were collected from hypertensive rats (35). 

Chronic renal failure is associated with uremia. Uremia accelerated EPC senescence by increasing ROS generation (36). Carbamylated darbepoetin (recombinant human erythropoietin) or darbepoetin (100 ng/ml) could decrease telomere shortening and senescence induced by TNF-α and uremic serum in EPCs from non-dialysis chronic kidney disease patients. The molecular mechanism was not declared (37).

In another *in vitro *study, nicotine (10 nM) increased telomerase activity through the PI3K/Akt pathway and reduced senescence in h-EPCs (38).

***In vivo studies***

Oral nicotine (100 ng/ml) intake after one month increased telomerase activity via Sirt1 up-regulation and decreased EPC senescence in mice. However, long-term treatment (3 and 6 months) had opposite results. The effect of nicotine was dependent on the signaling via nAChR-α7. Therefore, the expression of nAChR-α7 was increased in short-term exposure and decreased in long-term exposure due to a negative feedback mechanism (39). Sirt1 regulates the expression of TERT and anti-oxidant enzymes such as MnSOD through FoxO1 (40).

Beta-blockers, also known as beta-adrenergic blocking agents, such as celiprolol and atenolol, are anti-hypertensive drugs. Treating with celiprolol (50 mg/kg/day) for two weeks reduced EPC senescence by an anti-oxidant mechanism. It decreased mRNA expression of NADPH oxidase subunits in spontaneously hypertensive rats. In contrast, atenolol did not have such effects (41). In chronic diseases such as cardiovascular disease, it is preferable to select medicine with multiple therapeutic effects than other drugs in the same class.

In another study, atorvastatin (10 mg/kg, over three days) improved EPC senescence induced by indoxyl sulfate (a protein-bound uremic toxin) in acute kidney injury mice. Its protective mechanism was activated by increasing phosphorylated eNOS and reducing ROS. Indoxyl sulfate can increase ROS via the activation of NADPH oxidase (42).

Rivaroxaban (an anticoagulant drug, 1 or 3 mg/kg/day) enhanced the eNOS, Akt, and VEGF production. Also, it decreased the percentage of EPC senescence in the hyperglycemic condition in streptozotocin-induced diabetic mice (43).

***Human studies***

Healthy middle-aged male volunteers were treated with 0.4 mg/day of recombinant growth hormone (GH). The GH stimulated the IGF-1 related PI3K/Akt/eNOS signaling pathway, then elevated telomerase activity which in turn reduced senescence in their EPCs (44).

**Figure 1:**
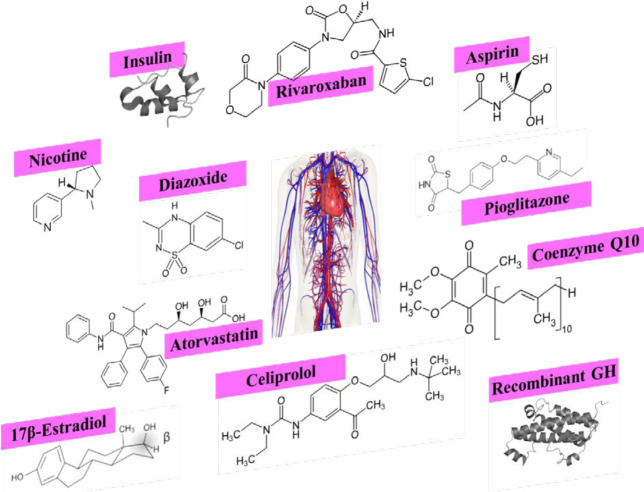
The chemical structure of protective agents against endothelial progenitor cell (EPC) senescence

Different conditions cause EPC senescence. We should consider EPC senescence in disease management and choose the agent with less adverse effects among one category of medicines for long-term use. Based on molecular pathways, we can find off-labeled indications for approved drugs to inhibit cellular senescence. We should also consider the time and dose-related impacts of therapeutic substances on cellular senescence in short and long-term prescriptions (as pointed out previously for nicotine). In some cases, A particular agent exhibits therapeutic outcomes in short-term or low-dose consumption but adverse outcomes in long-term or high-dose consumption. These reviewed medicines are potential candidates for the elderly population to enhance their EPC function and slow their cardiovascular aging process (Figure 1).

***Hematopoietic stem cells (HSCs)***

HSCs, characterized by expressing the tyrosine kinase receptor c-Kit (CD117) and the membrane glycoprotein Sca-1(c-Kit+ Sca-1+) without mature markers of Ter119, Gr-1, Mac-1, B220, CD4, and CD8, are located in BM. They have a high homing ability so that, even after transplantation, they can find their path to the recipient’s BM. They have an unlimited self-renewal ability for homeostasis and continuous blood cell turn-over throughout life, even after insults such as infection or therapeutic ablation (45). Studies have shown that HSCs infusion had positive outcomes in different conditions. HSCs infusion in the kidney recipients reduced transplantation failure and the required immunosuppressant dose (46). After chemotherapy, they alleviated rheumatoid arthritis in drug resistance patients (47). In diabetic patients, they improved various factors such as mean fasting blood sugar, postprandial blood sugar, and HbA1c (48). Experiments indicate that lead-acetate increased ROS and mitochondrial defect so that HSCs became senescence and their repopulation ability decreased (49). Ox-LDL increased oxidative stress and reduced telomerase activity which resulted in HSC senescence (50). Autologous HSC transplantation had a better result than allogeneic HSCs transplantation for superior immunosuppression in multiple sclerosis patients. HSCs reduced the number of new T2 lesions and decreased the annual relapse rate (51). Rapid telomere shortening in transplanted HSCs speeds up their senescence. Senescent HSCs have less clonal stability and homing ability (52). 

***In vitro studies***

Rapamycin (20 ng/ml) inhibited mTOR and increased Bmi1 expression. Hence it repressed p16 and senescence in the *ex-vivo* expansion of mice BM-HSCs (53). Bmi-1 cooperation with c-myc enhances telomerase activity which in turn decreases p16 and p19 expression, as well as cellular senescence (54). mTOR promotes cell proliferation and decreases autophagy while inhibits mitochondrial MnSOD and increases ROS levels (55). The dual effect of rapamycin through mTOR, senescence induction in EPSs, and senescence inhibition in HSCs, may be dose- or cell-dependent. So, it needs further investigation.

***In vivo studies***

In IR-induced long-term BM injury in a total-body irradiation mice model, treatment by 250 mg/kg/day of metformin (an anti-diabetic medicine) inhibited the expression of NOX4, as well as increased the cellular level of SOD1, SOD2, CAT, and GPX1 mRNAs thus ameliorated senescence in their HSCs (56). 

**Figure 2:**
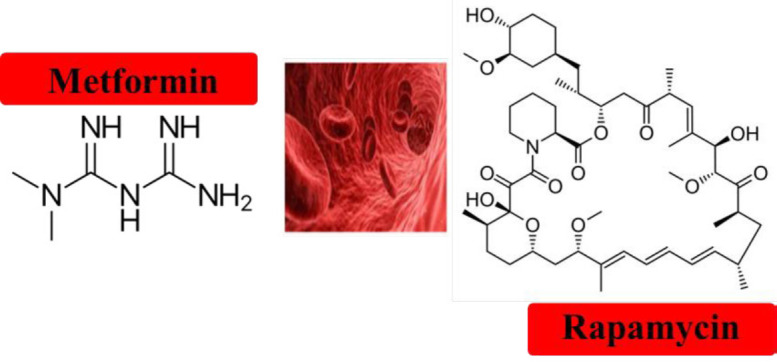
The chemical structure of protective agents against hematopoietic stem cell (HSC) senescence

These protective agents could be used as preventive or curative agents in cancer patients to reduce radiotherapy or chemotherapy adverse effects related to HSCs, such as susceptibility to infections. On the other hand, less HSC senescence during the pre-transplantation procedure can guarantee more procedure efficacy. Thus, *in vitro *pre-transplantation treatment or clinical prescription after transplantation can both improve HSC therapy (Figure 2). 

***Mesenchymal stem cell (MSCs)***

MSCs are located in various tissues and organs like BM, adipose tissue, umbilical cord blood, and Wharton Jelly, tendon, synovial, and blood circulation to maintain their homeostasis. MSCs in BM support hematopoietic stem cell niches. Moreover, they have shown immunomodulatory and anti-inflammatory activities. Their proliferation rate, however, is location-dependent, and senescence affects their homing ability toward injury or inflammatory lesions and multi-potency. Their immunophenotyping surface markers for purification are Stro1, CD73, and CD106 (57). MSC infusion has shown positive outcomes in clinical trials. Intra-articular injection of MSCs into the osteoarthritic knee improved function and pain of the knee joint (58). In addition, MSCs have been used in hepatitis B virus cirrhosis (59), Crohn’s disease (60), severe diabetic foot (61), amyotrophic lateral sclerosis (62), acute myocardial infarction (63), immunomodulation after liver transplantation (64), congestive heart failure (65), blood glucose control in type-2 diabetes (66), and multiple sclerosis (67). These studies have shown the safety of MSC therapy without adverse effects. The critical issue in such procedures is that MSCs before transplantation must be expanded to provide enough cells. However, in higher passages, replicative senescence happens. There is also an age-dependent increase in MSC senescence that result in impaired proliferation capacity (68). Senescent MSCs have shown morphological changes and low self-renewal potential (69). Furthermore, elderly patients do not have enough functional MSCs for efficient autologous transplantation (70).

Moreover, different diseases or chronic medicine therapies can induce MSC senescence. Heparin (anticoagulant agent) and most of the statins increased MSC senescence (71, 72). Patients with myelodysplastic syndrome have higher senescent MSCs, so they showed lower MSC proliferation ability (73). Chronic kidney disease caused premature MSC senescence and decreased regenerative potential in rats (74). The human immunodeficiency virus (HIV-1) p55-gag protein caused MSC senescence and decreased hematopoietic activity (75). High glucose via the Akt/mTOR signaling pathway induced MSC senescence (75). Senescent MSCs negatively impress their paracrine environment, immunomodulation, cell migration, differentiation, and therapeutic capability (76, 77). Their paracrine effect can change the tissue microenvironment. This change can trigger colon cancer cell growth (43) or induce progression and metastasis of breast cancer cells (78). So, preventing MSC senescence plays an essential role in cell therapy and cancer prevention. Since they are vastly distributed in the body, there is more concern about their senescence than other cells.

***In vitro studies***

Isosorbide dinitrate (a vasodilator medicine, 50 μM) attenuated high glucose-induced senescence in rat MSCs. This medicine increased mir-130b expression by reversing the down-regulation of ERK phosphorylation and FOXM1 expression. MiRNA-130b is downstream of the ERK/FOXM1 pathway. MiR-130b inhibited the expression of p21, although this observation needs further studies (79).

Rapamycin (100-500 nM) inhibited mTOR over-activation and senescence in the BM-MSCs of patients with systemic lupus erythematosus (80). 

Coenzyme Q 10 (10 or 100 µM) suppressed the expression of p21, p16, p53, as well as the Akt/mTOR signaling pathway, so inhibited senescence induced by D-Galactose in rat BM-MSCs (81). Idebenone (the analog of coenzyme Q10 for cardiovascular disease, 10, 20, or 30 μg/ml) progressed the cell cycle and delayed replicative senescence in rat MSCs (82).

Ascorbic acid (200 μM) inhibited ROS and Akt/mTOR signaling pathway in D-galactose-induced senescence in rat MSC (83).

1,25-dihydroxy vitamin D3 (100 nM) decreased p16 and delayed senescence in h-MSCs, however, it significantly increased ROS accumulation (84). 

Senescent MSCs are involved in the onset of osteoarthritis in menopausal women. 17β-estradiol (10^−12^ M) decreased senescence and improved the osteogenic ability of mini-pigs-BM-MSCs (85). Also, 17β-estradiol (10 ^-7^ M and 10 ^-9^ M) prevented telomere shortening by reducing oxidative stress and decreasing h-MSC senescence, but it did not affect p21 or SIRT1 protein expression (86). 

N-acetyl-cysteine (1 mM) decreased ROS, p38, p53, and senescence in BM-MSCs obtained from prolonged isolated thrombocytopenia (serious complication of allogeneic HSC transplantation) patients (87).

Uremic toxins in chronic kidney disease patients accelerate MSC senescence. Melatonin (100 μM) up-regulated PrPC and enhanced mitochondrial function in MSCs from CKD mice exposed to H_2_O_2_ and suppressed their senescence. Transplantation of these treated cells showed better activity for inhibiting necrosis and reducing the formation of collagen fibers in the ischemic area (88).

Melatonin (a natural hormone that regulates the biological clock and a sleeping pill, 10 nM, 1 μM, and 100 μM) decreased ROS levels, p53, p21, and p16. It also blocked the p53/ERK/p38 pathway and alleviated iron overload-induced senescence in mice BM-MSCs (89). Melatonin (10 nM, 1 μM, and 100 μM) up-regulated SIRT1 mRNA level, suppressed p16 and phosphorylated p38 expression, decreasing senescence in BM-MSCs exposed to H_2_O_2 _(56). 

Nicotinamide (a form of vitamin B_3_, 5 mM) delayed replicative senescence and improve differential ability in h-MSCs. Nicotinamide reduced ROS generation in mitochondria and increased cellular NAD+/NADH ratio and SIRT1 activation (90).

HIV protease inhibitor regimen (Atazanavir, Ritonavir, and lopinavir) has adverse effects on bone. H-MSCs exposed to atazanavir and lopinavir showed replicative senescence after 30 days. Pravastatin (25 μM) recovered SOD activity and ROS level in these cells and inhibited replicative senescence, improving their osteoblastic differentiation ability (91).

**Figure 3:**
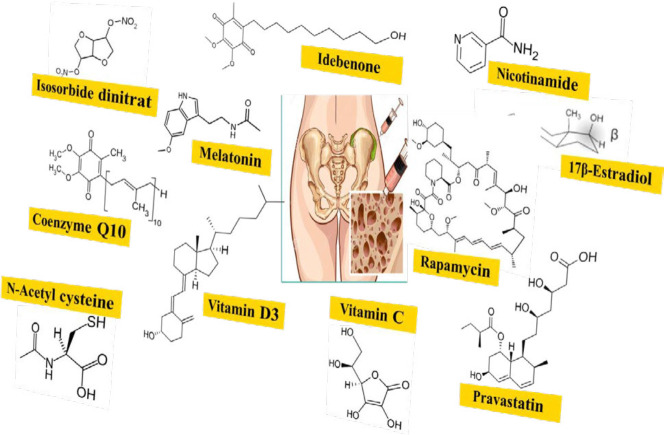
The chemical structure of protective agents against mesenchymal stem cell (MSC) senescence

Since MSCs are distributed in the whole body, they can be used as accessible stem cell sources. On the other hand, their senescent secretary inflammatory factors can impair many organs or trigger cancer formation, so MSC senescence inhibition is essential for preventative and curative intervention (Figure 3). 

***Adipose-derived mesenchymal stem cells (ASCs)***

Adipose-derived stem cells are a type of MSCs, and they have more abundant autologous sources, as well as rapid and high proliferation capacity than BM-MSCs. Also, they are cultured easier and show more genetic stability (92). Immunotolerance, cell surface molecular composition, and a high potential for multilineage differentiation of ASCs are similar to BM-MSCs (93). A comparison between BM-MSCs and ASCs of the same donors has shown that ASCs have a higher proliferation rate and a doubling population. ASCs kept their differentiation potential better than BM-MSCs in culture for a long time. Also, they expressed fewer senescence markers, so ASCs can be a good alternative for tissue reengineering (94). ASC injection could mitigate chronic pancreatitis (95), improved autonomic nervous system dysfunction in humans (96), improved multiple sclerosis (97), attenuated lungs and systemic injury induced by cigarette smoking (98)., and rescued early stages of diabetic retinopathy (99). The supernatant of ASCs culture ameliorated allergic airway inflammation through its immunomodulatory action (100) and improved wound healing (101) in clinical trials. 

The limitation of cell therapy with ASCs is cellular senescence (102). Besides this, highly distributed senescent adipose tissue in the body secretes inflammatory cytokines (103), increasing insulin resistance and obesity in metabolic syndrome disease (104). Furthermore, inflammatory IL-6 and IL-8 can produce more senescent cells (105).

***In vitro studies***

Vitamin C up-regulated Jhdm1a/b, c-Myc, Klf4, and down-regulated p21, so increased proliferation, postponed senescence, and transformation in higher passages of the mouse ASCs. Vitamin C did not have a significant effect on p53. Jhdm1a/b belongs to the Jumonji family proteins that are responsible for the demethylation of 3K36me2/3. Demethylated 3K36me2/3 can promote cell cycle progression. c-Myc and Klf4 have paramount roles in cell proliferation and differentiation, required for the self-renewal of ASCs (106). 

L-carnitine (a natural substance that helps the body with energy production, and, as a supplement sold in the market, 0.2 mM) decreased senescence by its anti-oxidant effects in rat ASCs. However, its detailed mechanism needs further investigation (107). 

Because of the increased use of the electromagnetic field for domestic and industrial purposes, different studies proved its influence on biological systems, including anti-oxidative enzymes, cell proliferation, and differentiation of stem cells. The electromagnetic field of 50 Hz and 20 milliTesla, could prevent the growth and metabolism of h-MSCs. ZnSO_4_ (0.14 μg/ml) increased TERT gene expression and decreased senescence in rat ASCs exposed to the highly low-frequency electromagnetic field of 50 Hz and 20 milliTesla (108). 

Melatonin (100 μM) protected h-ASCs against uremic toxin *p-*cresol-induced senescence (*p-*cresol found at high concentrations in the serum of CKD patients). Melatonin-induced phosphorylation of Akt activated the Akt signaling pathway, thus increased catalase activity and reduced ROS. Melatonin also decreased the level of phosphorylated mTOR by reducing AMPK. ROS elevation is strongly related to the AMPK activity. Melatonin could increase SMP30, which is involved in the anti-aging process (109). In another study, melatonin (10 μM), as an anti-oxidant, reduced replicative senescence in mice ASCs and preserved their differential potential. Melatonin in higher passages decreased NADPH oxidase content and, consequently ROS generation (110).

***In vivo studies***

ASCs isolated from mice treated with 2.8 mg/d of oral metformin for eight weeks had increased SOD activities and lessened ROS, NO, and cellular senescence. Metformin also enhanced its osteogenic properties and bone density (111). 

***Human studies***

In a study, diabetic kidney disease patients took oral administration of dasatinib (a tyrosine kinase inhibitor for leukemia, 100 mg) and quercetin (a plant pigment, 1000 mg) for three days. After eleven days, senescent and pre-senescent adipocyte progenitors in their bodies had decreased, and circulating inflammatory factors were removed (112).

**Figure 4:**
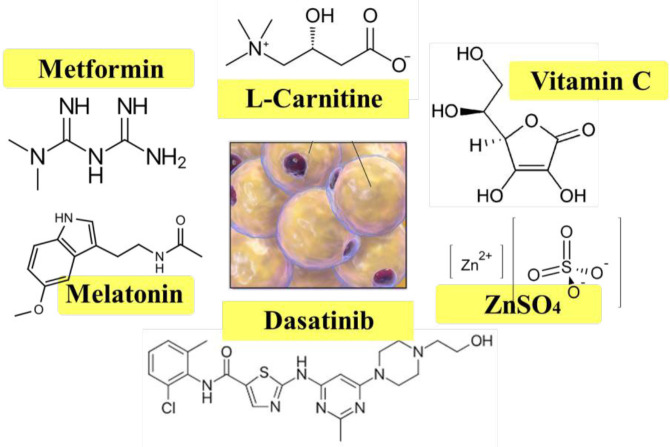
The chemical structure of protective agents against adipose-derived stem cell (ASC) senescence

Senescent cells are functionally impaired and, by releasing inflammatory molecules, create a harmful microenvironment for other cells, resulting in more senescent cells and finally damaged organs. Fat tissue is highly distributed in the body, so the prevention of ASC senescence via pharmacotherapy can attenuate different diseases related to obesity, such as insulin resistance and diabetes, and improve the quality of patients’ life. The inhibition of ASC senescence in cell culture is beneficial for harvesting more functional cells for cell therapy purposes (Figure 4). 

***Other adult stem cells***

*In vitro studies*

**Figure 5:**
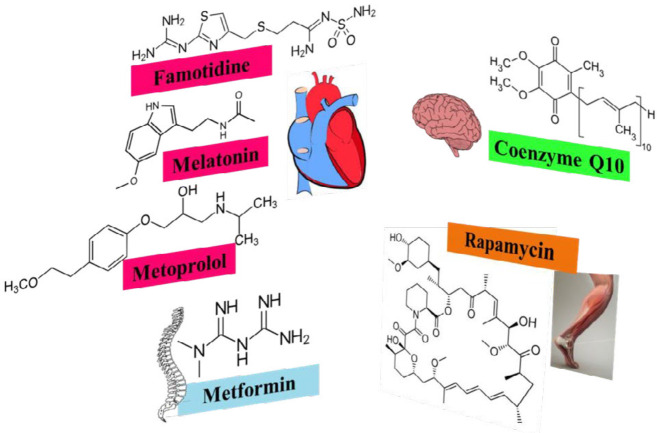
The chemical structure of protective agents against other adult stem cell senescence

Myeloblasts or muscle satellite cells are involved in muscle regeneration and aging declines their population. Rapamycin (10 nM) inhibited mTORC1, diminished senescence, and improved differentiation potential in muscle-derived stem/progenitor cells isolated from progeroid mice (113) (Figure 5).

Neural stem cells that generate new glial cells and neurons in the hippocampus and sub-ventricular zone of the lateral ventricles in the brain play an essential role in learning and memory (114). Anti-retroviral medicines (Tenofovir, Emtricitabine, Ritonavir, and Darunavir) increased ROS generation by mitochondria, induced telomere shortening, and decreased SIRT3 protein expression in mouse neural progenitor cells (NPCs). Mitochondria-targeted CoQ10 therapy (500 nM) reversed these effects and reduced cellular senescence (115).

Disc degeneration is an age-related disease associated with cellular senescence (116). It is one of the reasons for lower back pain and is very common in the elderly population (117). Metformin (1 mM) decreased LPS-induced-senescence and inflammatory factors in rabbit annulus fibrosus stem cells (118).

Cardiac stem cells (CSCs) and cardiac progenitor cells (CPCs) are involved in myocardial regeneration and repairment (119). Melatonin (10 μM) via long noncoding RNA H19 increased mir-675. Mir-675 overexpression down-regulated p21 and p53, which inhibited H_2_O_2_-induced senescence in CPCs, also reduced IL-6 secretion (120).

***In vivo studies***

Treating with famotidine (a histamine-2 receptor antagonist, 30 mg/kg/day) for two months improved self-renewal ability, proliferation capacity, and migration in CSCs of spontaneously hypertensive rats. This agent also decreased the ROS level and cellular senescence in these cells (121). However, the exact anti-oxidant mechanism was not investigated. Also, eating with metoprolol (50 mg/kg/d) for two months decreased ROS level and cellular senescence in CSCs of spontaneously hypertensive rats (122).

***Induced pluripotent stem cells (iPSCs)***

**Figure 6:**
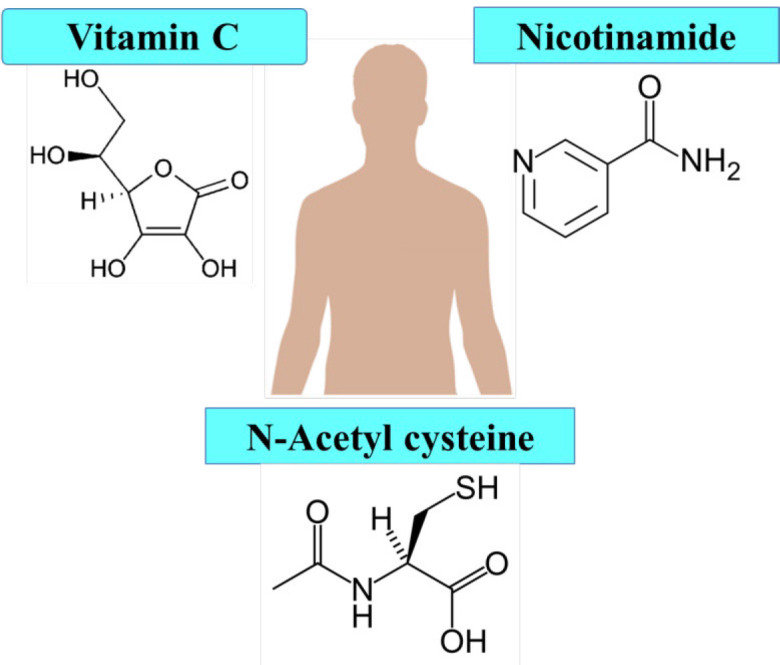
The chemical structure of protective agents against induced pluripotent stem cell senescence

Although adult stem cells seem to be useful for regeneration, their sources, differentiation, and expansion potential are limited. In addition to this, elderly patients have fewer stem cells than their younger donors. In contrast to adult stem cells, pluripotent stem cells, such as embryonic stem cells, have unlimited potential to proliferate and differentiate into all cell types (123). Various cells from different tissues can be reprogrammed to iPSCs then differentiated to other cell types. Since these cells are autologous, they are a preferable option for cell therapy (124, 125). There are different induction factors such as Oct4, Sox2, Klf4, c-Mys (OSKM) to reprogram adult stem cells into pluripotent stem cells. IPSCs provide a basis for personalized stem cell therapies and autologous transplantation, which does not have immunologic issues like transplant rejection (123). However, senescence is one of those key barriers to successful reprogramming (126) (Figure 6).

***In vitro studies***

N-acetylcysteine (1mM) alleviated oxidative stress and senescence in iPSCs and improved their hematopoietic differentiation (127). 

Vitamin C reduced p21, p53, and iPSCs senescence and improved their generation (128).

The OSKM gene induces ROS, p21, p53, p27, and p16 proteins during cell reprogramming. Nicotinamide (a biologically active amid of nicotinic acid, one mM) reversed these effects and inhibited senescence in human iPSCs (129). 

**Table 1:** The summary of protective mechanisms of medicines (*In vitro* studies)

Reference	Mechanisms	Cell source	Concentration	Agent
(66)	↓ mTOR ↑Bmi-1 ↓p16	Mice BM-HSCs	20 ng/ml	Rapamycin
(99)	↓ p16	h-MSC	100 nm	1,25-Dihydroxyvitamin D3
(98)	↓ROS ↓ Akt/mTOR signaling	rat BM-MSCs	200 μM	Ascorbic acid
(38)	Not studied	h-EPCs	50 -100 mM	Aspirin
(40)	↑Akt phosphorylation ↑Telomerase activity	h-EPCs	1 μM	Atorvastatin
(41)	↑PI3K/Akt ↓p27 ↑Cyclin A, cyclin F	h-EPCs	0. 1 μM	Atorvastatin Mevastatin
(50)	↑Telomere	non-dialysis stage 4–5 CKD patient EPCs	100 ng/ml	Carbamylated Darbepoetin or Darbepoetin
(96)	↓p21,p16,p53 ↓ Akt/mTOR signaling	rat BM-MSCs	10, or 100 𝜇m/L	Coenzyme Q10
(136)	↓ ROS ↑telomere length ↑SIRT3	mouse neural progenitor cells	500 nM	Coenzyme Q10
(97)	Progressed cell cycle	Rat MSCs	10, 20 or 30 μg/ml	Idebenone
(42)	↑ Akt phosphorylation ↑eNOS expression	Rat BM-EPCs	0. 1, 1 nM	Insulin
(94)	↑ERK/FOXM1 ↑ mir-130b	Rat MSCs	50 μM	Isosorbide dinitrate
(128)	Antioxidant	Rat ASCs	0.2 mM	L-Carnitine
(103)	↑ PrPC ↑ Mitochondrial function	CKD-Mice BM-MSCs	100 μM	Melatonin
(104)	↓ROS level ↓p53, p21,p16 ↓p53/ERK/p38	Mice BM-MSCs	10 nm, 1 μM and 100 μM	Melatonin
(70)	↑SIRT1 mRNA level ↓p16 ↓Phosphorylated p38	BM-MSCs	10 nm, 1 μM, and 100 μM	Melatonin
(130)	↑ AKT pathway ↑ catalase ↓ ROS AMPK/mTOR↓ SMP30↑	h-ASCs	100μM	Melatonin
(131)	↓ NADPH oxidase ↓ ROS	Mice ASCs	10 μM	Melatonin
(142)	↑long noncoding RNA H19 ↑mir-675 ↓p21, p53	CPCs	10 μM	Melatonin
(140)	↓inflammatory factors	Rabbit annulus fibrosus stem cells	1 mM	Metformin
(149)	Oxidative stress ↓	h-iPSCs	1 mM	N-Acetyl cysteine
(102)	↓ROS level ↓p53 ↓p38	h-MSCs	1mM	N-acetyl-cysteine
(105)	↓ROS level ↑NAD+/NADH ratio ↑SIRT1	h-MSCs	5 mM	Nicotinamide
(151)	↓ROS ↓p21,p53, p27 and p16	h-iPSCs	1 mM	Nicotinamide
(51)	↑ PI3K/Akt ↑Telomerase activity	h-EPCs	10 nM	Nicotine
(43)	↓Peroxynitrite ↓ Superoxide ↓AT1R ↑Telomerase activity	h-EPCs	10 μM	Pioglitazone
(106)	↑SOD ↓ROS level	h-MSCs	25 μM	Pravastatin
(95)	↓mTOR over-activation	BM-MSCs of Systemic lupus erythematosus patients	100-500 nM	Rapamycin
(134)	↓ mTORC1	Muscle stem cells of progeroid mice	10 nM	Rapamycin
(127)	↓p21	mice ASCs		Vitamin C
(150)	↓p21,p53	h-iPSCs		Vitamin c
(129)	↑TERT	Rat ASCs	0. 14 μg/ml	Znso4
(39)	↑AMPK phosphorylation ↑eNOS/Akt activity ↑HO-1 ↓ROS	h-EPCs	10 µM	Coenzyme Q 10
(46)	↑Telomerase activity ↓AT1R ↓NOX2 ↓Phosphorylation of p38/ MAPK	h-EPCs	100 nM	17β-Estradiol
(48)	↑Akt phosphorylation ↑Telomerase activity	rat BM-MNC EPCs	100 nM	17β-Estradiol
(100)	Not studied	Mini-pigs BM-MSCs	10^−^^12^ M	17β-Estradiol
(101)	↓ROS ↑Telomere	h-MSCs	10 ^_7^ and 10 ^_9^	17β-Estradiol

Akt: Cellular homolog of murine thymoma virus Akt8 oncoprotein, Ang2: Angiotensin-2, ASC: Adipose-drived stem cell, BM: Bone marrow, BP: Blood pressure, CAT: Catalase, CDKs: Cyclin-dependent protein kinases, CKD: Chronic kidney disease, DNA: Deoxyribonucleic acid, EGF: Epidermal growth factor, EPC: Endothelial progenitor cells, FBS: Fast blood sugar, FGF-2: Fibroblast growth factor-2, FOXO: Fork head transcription factor, GH: Growth hormone, GPX: Glutathione peroxidase, h-: Human-, HbA1c: Hemoglobin A1c, HO-1: Heme oxygenase-1, HPC: Hematopoietic progenitor cells, HSC: Hematopoietic stem cells, h TERT: Human telomerase reverse transcriptase, IGF-1: Insulin-like growth factor 1, IL: Interleukin, iPSC: Induced pluripotent stem cells, KSC: Keratinocyte stem cells, MAPK: p38 mitogen-activated protein kinase, MSC: Mesenchymal stem cells, Nox: NADPH oxidases, Nrf2: Nuclear factor erythroid 2–related factor 2, ox-LDL: Oxidized Low-Density Lipoprotein, PI3K: Phosphoinositide 3-kinase, PPAR γ: Peroxisome proliferator-activated receptor gamma, Rb: Retinoblastoma protein, ROS: Reactive oxygen species, SOD: Superoxide dismutase, t-BHP: Tert-butyl hydroperoxide, TERT: Human telomerase reverse transcriptase, TGF: Tumor growyh factor, UV: Ultraviolet

**Table 2:** The summary of protective mechanisms of medicines (*In vivo* and clinical studies)

**reference**	**Mechanisms**	**Cell source**	**Concentration**	**Agent**
(55)	↑ phosphorylated eNOS ↓ ROS	AKI mice EPCs	10 mg/kg for 3 days	Atorvastatin
(54)	↑ Antioxidant ↓NADPH oxidase subunits	Spontaneously hypertensive rats EPC	(50 mg/kg/day) For two weeks	Celiprolol
(143)	↓ROS	Spontaneously hypertensive rat CPCs	30mg/kg/d	Famotidine
(70)	NOX4↓ ↑SOD1, SOD2, CAT, GPX1	Mice HSCs	250 mg/kg/day	Metformin
(132)	↑SOD activity ↓ ROS, NO	Mice ASCs	2. 8mg/day oral for eight weeks	Metformin
(144)	↓ROS	Spontaneously hypertensive rat CPCs	50 mg/kg/d	Metoprolol
(52)	↑ Sirt 1 ↑ Telomerase activity	Mice EPCs	Oral 100 ng/ml	Nicotine
(56)	↑ eNOS ↑ Akt	STZ-induced diabetic mice EPCs	1 or 3mg/kg/day	Rivaroxaban
(133)	↓ Inflammatory factors	Clinical study: diabetic kidney disease patient Fat tissue biopsy	(100 mg) and (1000 mg) Oral administration for three days	Dasatinib and Quercetin
(57)	↑PI3K/Akt/eNOS ↑Telomerase activity	Clinical study: Healthy middle-aged male EPCs	0. 4 mg/d	Recombinant GH

Akt: Cellular homolog of murine thymoma virus Akt8 oncoprotein, Ang2: Angiotensin-2, ASC: Adipose-drived stem cell, BM: Bone marrow, BP: Blood pressure, CAT: Catalase, CDKs: Cyclin-dependent protein kinases, CKD: Chronic kidney disease, DNA: Deoxyribonucleic acid, EGF: Epidermal growth factor, EPC: Endothelial progenitor cells, FBS: Fast blood sugar, FGF-2: Fibroblast growth factor-2, FOXO: Fork head transcription factor, GH: Growth hormone, GPX: Glutathione peroxidase, h-: Human-, HbA1c: Hemoglobin A1c, HO-1: Heme oxygenase-1, HPC: Hematopoietic progenitor cells, HSC: Hematopoietic stem cells, h TERT: Human telomerase reverse transcriptase, IGF-1: Insulin-like growth factor 1, IL: Interleukin, iPSC: Induced pluripotent stem cells, KSC: Keratinocyte stem cells, MAPK: p38 mitogen-activated protein kinase, MSC: Mesenchymal stem cells, Nox: NADPH oxidases, Nrf2: Nuclear factor erythroid 2–related factor 2, ox-LDL: Oxidized Low-Density Lipoprotein, PI3K: Phosphoinositide 3-kinase, PPAR γ: Peroxisome proliferator-activated receptor gamma, Rb: Retinoblastoma protein, ROS: Reactive oxygen species, SOD: Superoxide dismutase, t-BHP: Tert-butyl hydroperoxide, TERT: Human telomerase reverse transcriptase, TGF: Tumor growyh factor, UV: Ultraviolet

## Discussion

Stem cell senescence has been studied in aging, diseases, adverse drug effects, and as a challenging phenomenon in cell therapy. The most investigated types of these cells are EPCs, HSCs, and MSCs. Other investigated kinds include CPCs, myeloblasts, and iPSCs. EPCs are involved in vascular homeostasis and new blood vessel regeneration (130). The decrease in their functional cell number is associated with aging and atherosclerotic processes (20). HSCs are involved in blood coagulation, oxygen transportation, and immune system function, so their senescence leads to blood dysfunction (52). MSCs exist in many tissues, including bone marrow, adipose tissue, the bloodstream, and cord blood (57). MSCs have high self-renewal capacity and the ability to differentiate into other kinds of cells, such as adipocytes, chondrocytes, and osteoblasts, depending on their host organ (131). Although adult stem cells appear to be valuable sources for regeneration, they have limited sources, differentiation, and expansion potential (123). However, differentiated cells can be reprogrammed to iPSCs and then differentiated to desired cell types (124, 125). 

As reviewed in this paper, most of these protective agents increased telomerase activity or decreased oxidative damage via various anti-oxidant mechanisms, which ultimately inhibited cellular senescence.

Senescence prevention in the body results in health and longevity. Various medicines inhibit senescence through different mechanisms. As mentioned in this review, 17β-estradiol, melatonin, metformin, rapamycin, coenzyme Q10, N-acetyl cysteine, and vitamin C were the most studied agents in different kinds of stem cells (Figure1). Although most of these studies were *in vitro, *we can consider these agents in cell therapy to increase the shelf life and the functional cell number of donated stem cells before transplantation to achieve more clinical success. Moreover, *in vitro* studies are the first step towards clinical studies. Although more studies are necessary for clinical application, these reviewed agents have been used in the clinical setting for different purposes for a long time; therefore, we only need to evaluate their systemic anti-senescence effects and effective anti-senescence dosages. 

## Conclusion

Off-label use of approved medicines and supplements is a convenient, safe, and economical approach to prevent stem cell senescence both *in vitro* and *in vivo*. These agents provide a wide range of options based on targeted cells. Since all of them have passed substantial safety trials, we only need to determine their effective dosage to prevent stem cell senescence. Maybe it seems that heterogeneity of administration, patients, and diseases, can make repurposing inefficient and time-consuming. Still, in comparison with discovering new anti-senescence agents, this approach is much more economical and accessible. Moreover, performing retrospective studies for each medicine can address these issues.

## References

[1] Mohammadi E, Mehri S, Badie Bostan H, Hosseinzadeh H (2018). Protective effect of crocin against d-galactose-induced aging in mice. Avicenna J Phytomed.

[2] Rodier F, Campisi J (2011). Four faces of cellular senescence. J Cell Biol.

[3] Sharpless NE, DePinho RA (2004). Telomeres, stem cells, senescence, and cancer. J Clin Invest.

[4] Flach J, Bakker ST, Mohrin M, Conroy PC, Pietras EM, Reynaud D (2014). Replication stress is a potent driver of functional decline in ageing haematopoietic stem cells. Nature.

[5] Signer RAJ, Morrison SJ (2013). Mechanisms that Regulate Stem Cell Aging and Life Span. Cell Stem Cell.

[6] Lee AC, Fenster BE, Ito H, Takeda K, Bae NS, Hirai T (1999). Ras proteins induce senescence by altering the intracellular levels of reactive oxygen species. J Biol Chem.

[7] Shin DH, Lee SJ, Kim JS, Ryu JH, Kim JS (2015). Synergistic effect of immunoliposomal gemcitabine and bevacizumab in glioblastoma stem cell-targeted therapy. J Biomed Nanotechnol.

[8] Childs BG, Durik M, Baker DJ, van Deursen JM (2015). Cellular senescence in aging and age-related disease: from mechanisms to therapy. Nat Med.

[9] Korski KI, Kubli DA, Wang BJ, Khalafalla FG, Monsanto MM, Firouzi F (2019). Hypoxia prevents mitochondrial dysfunction and senescence in human c-Kit(+) cardiac progenitor cells. Stem Cells.

[10] Liu S, Mahairaki V, Bai H, Ding Z, Li J, Witwer KW (2019). Highly purified human extracellular vesicles produced by stem cells alleviate aging cellular phenotypes of senescent human cells. Stem Cells.

[11] Rao VV, Vu MK, Ma H, Killaars AR, Anseth KS (2019). Rescuing mesenchymal stem cell regenerative properties on hydrogel substrates post serial expansion. Bioeng Transl Med.

[12] Qi L, Wang R, Shi Q, Yuan M, Jin M, Li D (2019). Umbilical cord mesenchymal stem cell conditioned medium restored the expression of collagen II and aggrecan in nucleus pulposus mesenchymal stem cells exposed to high glucose. J Bone Miner Metab.

[13] Vono R, Jover Garcia E, Spinetti G, Madeddu P (2018). Oxidative stress in mesenchymal stem cell senescence: Regulation by coding and noncoding RNAs. Antioxid Redox Signal.

[14] Khatiwala RV, Zhang S, Li X, Devejian N, Bennett E, Cai C (2018). Inhibition of p16(INK4A) to rejuvenate aging human cardiac progenitor cells via the upregulation of anti-oxidant and NFkappaB signal pathways. Stem Cell Rev Rep.

[15] Tousian H, Razavi BM, Hosseinzadeh H (2020). Looking for immortality: Review of phytotherapy for stem cell senescence. Iran J Basic Med Sci.

[16] Arbab AS, Bashaw LA, Miller BR, Jordan EK, Bulte JW, Frank JA (2003). Intracytoplasmic tagging of cells with ferumoxides and transfection agent for cellular magnetic resonance imaging after cell transplantation: methods and techniques. Transplantation.

[17] Hristov M, Weber C (2004). Endothelial progenitor cells: characterization, pathophysiology, and possible clinical relevance. J Cell Mol Med.

[18] Hill JM, Zalos G, Halcox JP, Schenke WH, Waclawiw MA, Quyyumi AA (2003). Circulating endothelial progenitor cells, vascular function, and cardiovascular risk. N Engl J Med.

[19] Tobler K, Freudenthaler A, Baumgartner-Parzer SM, Wolzt M, Ludvik B, Nansalmaa E (2010). Reduction of both number and proliferative activity of human endothelial progenitor cells in obesity. Int J Obes (Lond).

[20] Scheubel RJ, Zorn H, Silber RE, Kuss O, Morawietz H, Holtz J (2003). Age-dependent depression in circulating endothelial progenitor cells in patients undergoing coronary artery bypass grafting. J Am Coll Cardiol.

[21] Lee ST, Chu K, Jung KH, Kim DH, Kim EH, Choe VN (2008). Decreased number and function of endothelial progenitor cells in patients with migraine. Neurology.

[22] Kobayashi K, Imanishi T, Akasaka T (2006). Endothelial progenitor cell differentiation and senescence in an angiotensin II-infusion rat model. Hypertens Res.

[23] De Falco E, Carnevale R, Pagano F, Chimenti I, Fianchini L, Bordin A (2016). Role of NOX2 in mediating doxorubicin-induced senescence in human endothelial progenitor cells. Mech Ageing Dev.

[24] Sugawara J, Mitsui-Saito M, Hayashi C, Hoshiai T, Senoo M, Chisaka H (2005). Decrease and senescence of endothelial progenitor cells in patients with preeclampsia. J Clin Endocrinol Metab.

[25] Hu Z, Zhang F, Yang Z, Zhang J, Zhang D, Yang N (2008). Low-dose aspirin promotes endothelial progenitor cell migration and adhesion and prevents senescence. Cell Biol Int.

[26] Tsai H-Y, Lin C-P, Huang P-H, Li S-Y, Chen J-S, Lin F-Y (2016). Coenzyme Q10 attenuates high glucose-induced endothelial progenitor cell dysfunction through AMP-Activated protein kinase pathways. J Diabetes Res.

[27] Zhu JH, Chen JZ, Wang XX, Xie XD, Sun J, Zhang FR (2006). Homocysteine accelerates senescence and reduces proliferation of endothelial progenitor cells. J Mol Cell Cardiol.

[28] Assmus B, Urbich C, Aicher A, Hofmann WK, Haendeler J, Rossig L (2003). HMG-CoA reductase inhibitors reduce senescence and increase proliferation of endothelial progenitor cells via regulation of cell cycle regulatory genes. Circ Res.

[29] Zhao L, Cao F, Yin T, Sun D, Cheng K, Zhang J (2011). Moderate dose insulin promotes function of endothelial progenitor cells. Cell Biol Int.

[30] Imanishi T, Kobayashi K, Kuroi A, Ikejima H, Akasaka T (2008). Pioglitazone inhibits angiotensin II-induced senescence of endothelial progenitor cell. Hypertens Res.

[31] Shen X, Wang M, Bi X, Zhang J, Wen S, Fu G (2016). Resveratrol prevents endothelial progenitor cells from senescence and reduces the oxidative reaction via PPARgamma/HO1 pathways. Mol Med Rep.

[32] Alhurani RE, Chahal CAA, Ahmed AT, Mohamed EA, Miller VM (2016). Sex hormone therapy and progression of cardiovascular disease in menopausal women. Clin Sci (Lond).

[33] Imanishi T, Hano T, Nishio I (2005). Estrogen reduces angiotensin II-induced acceleration of senescence in endothelial progenitor cells. Hypertens Res.

[34] Tormos AM, Talens-Visconti R, Nebreda AR, Sastre J (2013). p38 MAPK: a dual role in hepatocyte proliferation through reactive oxygen species. Free Radic Res.

[35] Imanishi T, Hano T, Nishio I (2005). Estrogen reduces endothelial progenitor cell senescence through augmentation of telomerase activity. J Hypertens.

[36] D'Apolito M, Colia AL, Lasalvia M, Capozzi V, Falcone MP, Pettoello-Mantovani M (2017). Urea-induced ROS accelerate senescence in endothelial progenitor cells. Atherosclerosis.

[37] Ramirez R, Carracedo J, Nogueras S, Buendia P, Merino A, Canadillas S (2009). Carbamylated darbepoetin derivative prevents endothelial progenitor cell damage with no effect on angiogenesis. J Mol Cell Cardiol.

[38] Junhui Z, Xiaojing H, Binquan Z, Xudong X, Junzhu C, Guosheng F (2009). Nicotine-reduced endothelial progenitor cell senescence through augmentation of telomerase activity via the PI3K/Akt pathway. Cytotherapy.

[39] You J, Sun J, Ma T, Yang Z, Wang X, Zhang Z (2017). Curcumin induces therapeutic angiogenesis in a diabetic mouse hindlimb ischemia model via modulating the function of endothelial progenitor cells. Stem Cell Res Ther.

[40] Makino N, Oyama J, Maeda T, Koyanagi M, Higuchi Y, Shimokawa I (2016). FoxO1 signaling plays a pivotal role in the cardiac telomere biology responses to calorie restriction. Mol Cell Biochem.

[41] Yao E-H, Fukuda N, Matsumoto T, Katakawa M, Yamamoto C, Han Y (2008). Effects of the antioxidative β-Blocker celiprolol on endothelial progenitor cells in hypertensive rats. Am J Hypertens.

[42] Wu VC, Young GH, Huang PH, Lo SC, Wang KC, Sun CY (2013). In acute kidney injury, indoxyl sulfate impairs human endothelial progenitor cells: modulation by statin. Angiogenesis.

[43] Li Y, Xu X, Wang L, Liu G, Li Y, Wu X (2015). Senescent mesenchymal stem cells promote colorectal cancer cells growth via galectin-3 expression. Cell Biosci.

[44] Thum T, Hoeber S, Froese S, Klink I, Stichtenoth DO, Galuppo P (2007). Age-dependent impairment of endothelial progenitor cells is corrected by growth-hormone-mediated increase of insulin-like growth-factor-1. Circ Res.

[45] Rossi L, Challen GA, Sirin O, Lin KK-Y, Goodell MA (2011). Hematopoietic stem cell characterization and isolation. Methods Mol Biol.

[46] Vanikar AV, Trivedi HL, Kumar A, Gopal SC, Patel HV, Gumber MR (2014). Co-infusion of donor adipose tissue-derived mesenchymal and hematopoietic stem cells helps safe minimization of immunosuppression in renal transplantation - single center experience. Ren Fail.

[47] Verburg RJ, Kruize AA, van den Hoogen FH, Fibbe WE, Petersen EJ, Preijers F (2001). High-dose chemotherapy and autologous hematopoietic stem cell transplantation in patients with rheumatoid arthritis: results of an open study to assess feasibility, safety, and efficacy. Arthritis Rheum.

[48] Thakkar UG, Trivedi HL, Vanikar AV, Dave SD (2015). Insulin-secreting adipose-derived mesenchymal stromal cells with bone marrow-derived hematopoietic stem cells from autologous and allogenic sources for type 1 diabetes mellitus. Cytotherapy.

[49] Liu J, Jia DY, Cai SZ, Li CP, Zhang MS, Zhang YY (2015). Mitochondria defects are involved in lead-acetate-induced adult hematopoietic stem cell decline. Toxicol Lett.

[50] Zhang XP, Zhang GH, Wang YY, Liu J, Wei Q, Xu CY (2013). Oxidized low-density lipoprotein induces hematopoietic stem cell senescence. Cell Biol Int.

[51] Mancardi GL, Sormani MP, Gualandi F, Saiz A, Carreras E, Merelli E (2015). Autologous hematopoietic stem cell transplantation in multiple sclerosis: a phase II trial. Neurology.

[52] Chen J (2011). Hematopoietic stem cell development, aging and functional failure. Int J Hematol.

[53] Luo Y, Li L, Zou P, Wang J, Shao L, Zhou D (2014). Rapamycin enhances long-term hematopoietic reconstitution of ex vivo expanded mouse hematopoietic stem cells by inhibiting senescence. Transplantation.

[54] Molofsky AV, He S, Bydon M, Morrison SJ, Pardal R (2005). Bmi-1 promotes neural stem cell self-renewal and neural development but not mouse growth and survival by repressing the p16(Ink4a) and p19(Arf) senescence pathways. Genes Dev.

[55] Xu S, Cai Y, Wei Y (2014). mTOR Signaling from Cellular Senescence to Organismal Aging. Aging Dis.

[56] Xu G, Wu H, Zhang J, Li D, Wang Y, Wang Y (2015). Metformin ameliorates ionizing irradiation-induced long-term hematopoietic stem cell injury in mice. Free Radic Biol Med.

[57] Stewart MC, Stewart AA (2011). Mesenchymal stem cells: characteristics, sources, and mechanisms of action. Vet Clin North Am Equine Pract.

[58] Jo CH, Lee YG, Shin WH, Kim H, Chai JW, Jeong EC (2014). Intra-articular injection of mesenchymal stem cells for the treatment of osteoarthritis of the knee: a proof-of-concept clinical trial. Stem Cells.

[59] Zheng G, Huang L, Tong H, Shu Q, Hu Y, Ge M (2014). Treatment of acute respiratory distress syndrome with allogeneic adipose-derived mesenchymal stem cells: a randomized, placebo-controlled pilot study. Respir Res.

[60] Panes J, Garcia-Olmo D, Van Assche G, Colombel JF, Reinisch W, Baumgart DC (2016). Expanded allogeneic adipose-derived mesenchymal stem cells (Cx601) for complex perianal fistulas in Crohn's disease: a phase 3 randomised, double-blind controlled trial. Lancet.

[61] Qin HL, Zhu XH, Zhang B, Zhou L, Wang WY (2016). Clinical evaluation of human umbilical cord mesenchymal stem cell transplantation after angioplasty for diabetic foot. Exp Clin Endocrinol Diabetes.

[62] Rushkevich YN, Kosmacheva SM, Zabrodets GV, Ignatenko SI, Goncharova NV, Severin IN (2015). The use of autologous mesenchymal stem cells for cell therapy of patients with amyotrophic lateral sclerosis in belarus. Bull Exp Biol Med.

[63] Gao LR, Chen Y, Zhang NK, Yang XL, Liu HL, Wang ZG (2015). Intracoronary infusion of Wharton's jelly-derived mesenchymal stem cells in acute myocardial infarction: double-blind, randomized controlled trial. BMC Med.

[64] Soeder Y, Loss M, Johnson CL, Hutchinson JA, Haarer J, Ahrens N (2015). First-in-human case study: Multipotent adult progenitor cells for immunomodulation after liver transplantation. Stem Cells Transl Med.

[65] Zhao XF, Xu Y, Zhu ZY, Gao CY, Shi YN (2015). Clinical observation of umbilical cord mesenchymal stem cell treatment of severe systolic heart failure. Genet Mol Res.

[66] Kong D, Zhuang X, Wang D, Qu H, Jiang Y, Li X (2014). Umbilical cord mesenchymal stem cell transfusion ameliorated hyperglycemia in patients with type 2 diabetes mellitus. Clin Lab.

[67] Llufriu S, Sepulveda M, Blanco Y, Marin P, Moreno B, Berenguer J (2014). Randomized placebo-controlled phase II trial of autologous mesenchymal stem cells in multiple sclerosis. PLoS One.

[68] Zhou S, Greenberger JS, Epperly MW, Goff JP, Adler C, Leboff MS (2008). Age-related intrinsic changes in human bone-marrow-derived mesenchymal stem cells and their differentiation to osteoblasts. Aging Cell.

[69] Lee JK, Jin HK, Endo S, Schuchman EH, Carter JE, Bae JS (2010). Intracerebral transplantation of bone marrow-derived mesenchymal stem cells reduces amyloid-beta deposition and rescues memory deficits in Alzheimer's disease mice by modulation of immune responses. Stem Cells.

[70] Jaiswal N, Haynesworth SE, Caplan AI, Bruder SP (1997). Osteogenic differentiation of purified, culture-expanded human mesenchymal stem cells in vitro. J Cell Biochem.

[71] Izadpanah R, Schachtele DJ, Pfnur AB, Lin D, Slakey DP, Kadowitz PJ (2015). The impact of statins on biological characteristics of stem cells provides a novel explanation for their pleiotropic beneficial and adverse clinical effects. Am J Physiol Cell Physiol.

[72] Ling L, Camilleri ET, Helledie T, Samsonraj RM, Titmarsh DM, Chua RJ (2016). Effect of heparin on the biological properties and molecular signature of human mesenchymal stem cells. Gene.

[73] Liu Q, Zhu H, Dong J, Li H, Zhang H (2015). Defective proliferative potential of MSCs from pediatric myelodysplastic syndrome patients is associated with cell senescence. Int J Clin Exp Pathol.

[74] Klinkhammer BM, Kramann R, Mallau M, Makowska A, van Roeyen CR, Rong S (2014). Mesenchymal stem cells from rats with chronic kidney disease exhibit premature senescence and loss of regenerative potential. PLoS One.

[75] Zhang D, Lu H, Chen Z, Wang Y, Lin J, Xu S (2017). High glucose induces the aging of mesenchymal stem cells via Akt/mTOR signaling. Mol Med Rep.

[76] Wang Y, Han ZB, Song YP, Han ZC (2012). Safety of mesenchymal stem cells for clinical application. Stem Cells Int.

[77] Despars G, Carbonneau CL, Bardeau P, Coutu DL, Beauséjour CM (2013). Loss of the osteogenic differentiation potential during senescence is limited to bone progenitor cells and is dependent on p53. PLoS One.

[78] Di GH, Liu Y, Lu Y, Liu J, Wu C, Duan HF (2014). IL-6 secreted from senescent mesenchymal stem cells promotes proliferation and migration of breast cancer cells. PLoS One.

[79] Xu J, Huang Z, Lin L, Fu M, Song Y, Shen Y (2015). miRNA-130b is required for the ERK/FOXM1 pathway activation-mediated protective effects of isosorbide dinitrate against mesenchymal stem cell senescence induced by high glucose. Int J Mol Med.

[80] Gu Z, Tan W, Ji J, Feng G, Meng Y, Da Z (2016). Rapamycin reverses the senescent phenotype and improves immuno-regulation of mesenchymal stem cells from MRL/lpr mice and systemic lupus erythematosus patients through inhibition of the mTOR signaling pathway. Aging (Albany NY).

[81] Zhang D, Yan B, Yu S, Zhang C, Wang B, Wang Y (2015). Coenzyme Q10 inhibits the aging of mesenchymal stem cells induced by D-galactose through Akt/mTOR signaling. Oxid Med Cell Longev.

[82] Zhang J, Zhang J, Li T, Zhang N, Tang S, Tao Z (2018). Effect of idebenone on bone marrow mesenchymal stem cells in vitro. Mol Med Rep.

[83] Yang M, Teng S, Ma C, Yu Y, Wang P, Yi C (2018). Ascorbic acid inhibits senescence in mesenchymal stem cells through ROS and AKT/mTOR signaling. Cytotechnology.

[84] Klotz B, Mentrup B, Regensburger M, Zeck S, Schneidereit J, Schupp N (2012). 1,25-dihydroxyvitamin D3 treatment delays cellular aging in human mesenchymal stem cells while maintaining their multipotent capacity. PLoS One.

[85] Lee WJ, Lee SC, Lee JH, Rho GJ, Lee SL (2016). Differential regulation of senescence and in vitro differentiation by 17beta-estradiol between mesenchymal stem cells derived from male and female mini-pigs. J Vet Sci.

[86] Breu A, Sprinzing B, Merkl K, Bechmann V, Kujat R, Jenei-Lanzl Z (2011). Estrogen reduces cellular aging in human mesenchymal stem cells and chondrocytes. J Orthop Res.

[87] Kong Y, Song Y, Tang FF, Zhao HY, Chen YH, Han W (2018). N-acetyl-L-cysteine improves mesenchymal stem cell function in prolonged isolated thrombocytopenia post-allotransplant. Br J Haematol.

[88] Han YS, Kim SM, Lee JH, Jung SK, Noh H, Lee SH (2019). Melatonin protects chronic kidney disease mesenchymal stem cells against senescence via PrP(C) -dependent enhancement of the mitochondrial function. J Pineal Res.

[89] Yang F, Yang L, Li Y, Yan G, Feng C, Liu T (2017). Melatonin protects bone marrow mesenchymal stem cells against iron overload-induced aberrant differentiation and senescence. J Pineal Res.

[90] Ok JS, Song SB, Hwang ES (2018). Enhancement of replication and differentiation potential of human bone marrow stem cells by nicotinamide treatment. Int J Stem Cells.

[91] Hernandez-Vallejo SJ, Beaupere C, Larghero J, Capeau J, Lagathu C (2013). HIV protease inhibitors induce senescence and alter osteoblastic potential of human bone marrow mesenchymal stem cells: beneficial effect of pravastatin. Aging Cell.

[92] Meza-Zepeda LA, Noer A, Dahl JA, Micci F, Myklebost O, Collas P (2008). High-resolution analysis of genetic stability of human adipose tissue stem cells cultured to senescence. J Cell Mol Med.

[93] Gm C, El L, Jj C, Mr B, Ge DC, Ma S (2011). Direct comparison of progenitor cells derived from adipose, muscle, and bone marrow from wild-type or craniosynostotic rabbits. Plast Reconstr Surg.

[94] Burrow KL, Hoyland JA, Richardson SM (2017). Human adipose-derived stem cells exhibit enhanced proliferative capacity and retain multipotency longer than donor-matched bone marrow mesenchymal stem cells during expansion in vitro. Stem Cells Int.

[95] Sun Z, Gou W, Kim DS, Dong X, Strange C, Tan Y (2017). Adipose stem cell therapy mitigates chronic pancreatitis via differentiation into acinar-like cells in mice. Mol Ther.

[96] Numan MT, Kamdar A, Young J, Butler IJ (2017). Autologous adipose stem cell therapy for autonomic nervous system dysfunction in two young patients. Stem Cells Dev.

[97] Stepien A, Dabrowska NL, Maciagowska M, Macoch RP, Zolocinska A, Mazur S (2016). Clinical application of autologous adipose stem cells in patients with multiple sclerosis: preliminary results. Mediators Inflamm.

[98] Schweitzer KS, Johnstone BH, Garrison J, Rush NI, Cooper S, Traktuev DO (2011). Adipose stem cell treatment in mice attenuates lung and systemic injury induced by cigarette smoking. Am J Respir Crit Care Med.

[99] Rajashekhar G, Ramadan A, Abburi C, Callaghan B, Traktuev DO, Evans-Molina C (2014). Regenerative therapeutic potential of adipose stromal cells in early stage diabetic retinopathy. PLoS One.

[100] Yu HS, Park MK, Kang SA, Cho KS, Mun SJ, Roh HJ (2017). Culture supernatant of adipose stem cells can ameliorate allergic airway inflammation via recruitment of CD4+CD25+Foxp3 T cells. Stem Cell Res Ther.

[101] Zhao J, Hu L, Liu J, Gong N, Chen L (2013). The effects of cytokines in adipose stem cell-conditioned medium on the migration and proliferation of skin fibroblasts in vitro. Biomed Res Int.

[102] Mantovani C, Terenghi G, Magnaghi V (2014). Senescence in adipose-derived stem cells and its implications in nerve regeneration. Neural Regen Res.

[103] Minamino T, Orimo M, Shimizu I, Kunieda T, Yokoyama M, Ito T (2009). A crucial role for adipose tissue p53 in the regulation of insulin resistance. Nat Med.

[104] Hotamisligil GS, Shargill NS, Spiegelman BM (1993). Adipose expression of tumor necrosis factor-alpha: direct role in obesity-linked insulin resistance. Science.

[105] Orjalo AV, Bhaumik D, Gengler BK, Scott GK, Campisi J (2009). Cell surface-bound IL-1alpha is an upstream regulator of the senescence-associated IL-6/IL-8 cytokine network. Proc Natl Acad Sci U S A.

[106] Wei C, Liu X, Tao J, Wu R, Zhang P, Bian Y (2014). Effects of vitamin C on characteristics retaining of in vitro-cultured mouse adipose-derived stem cells. In Vitro Cell Dev Biol Anim.

[107] Mobarak H, Fathi E, Farahzadi R, Zarghami N, Javanmardi S (2017). L-carnitine significantly decreased aging of rat adipose tissue-derived mesenchymal stem cells. Vet Res Commun.

[108] Fathi E, Farahzadi R, Rahbarghazi R, Samadi Kafil H, Yolmeh R (2017). Rat adipose-derived mesenchymal stem cells aging reduction by zinc sulfate under extremely low frequency electromagnetic field exposure is associated with increased telomerase reverse transcriptase gene expression. Vet Res Forum.

[109] Yun SP, Han YS, Lee JH, Kim SM, Lee SH (2017). Melatonin rescues mesenchymal stem cells from senescence induced by the uremic toxin p-cresol via inhibiting mTOR-dependent autophagy. Biomol Ther (Seoul).

[110] Liao N, Shi Y, Zhang C, Zheng Y, Wang Y, Zhao B (2019). Anti-oxidants inhibit cell senescence and preserve stemness of adipose tissue-derived stem cells by reducing ROS generation during long-term in vitro expansion. Stem Cell Res Ther.

[111] Marycz K, Tomaszewski KA, Kornicka K, Henry BM, Wronski S, Tarasiuk J (2016). Metformin decreases reactive oxygen species, enhances osteogenic properties of adipose-derived multipotent mesenchymal stem cells in vitro, and increases bone density in vivo. Oxid Med Cell Longev.

[112] Hickson LJ, Langhi Prata LGP, Bobart SA, Evans TK, Giorgadze N, Hashmi SK (2019). Senolytics decrease senescent cells in humans: Preliminary report from a clinical trial of Dasatinib plus Quercetin in individuals with diabetic kidney disease. EBioMedicine.

[113] Kawakami Y, Hambright WS, Takayama K, Mu X, Lu A, Cummins JH (2019). Rapamycin rescues age-related changes in muscle-derived stem/progenitor cells from progeroid mice. Mol Ther Methods Clin Dev.

[114] Galli R, Gritti A, Bonfanti L, Vescovi AL (2003). Neural stem cells: an overview. Circ Res.

[115] Velichkovska M, Surnar B, Nair M, Dhar S, Toborek M (2019). Targeted mitochondrial COQ10 delivery attenuates antiretroviral-drug-induced senescence of neural progenitor cells. Mol Pharm.

[116] Sakai D, Schol J (2017). Cell therapy for intervertebral disc repair: Clinical perspective. J Orthop Translat.

[117] Molinos M, Almeida CR, Caldeira J, Cunha C, Goncalves RM, Barbosa MA (2015). Inflammation in intervertebral disc degeneration and regeneration. J R Soc Interface.

[118] Han Y, Yuan F, Deng C, He F, Zhang Y, Shen H (2019). Metformin decreases LPS-induced inflammatory response in rabbit annulus fibrosus stem/progenitor cells by blocking HMGB1 release. Aging (Albany NY).

[119] Itzhaki-Alfia A, Leor J, Raanani E, Sternik L, Spiegelstein D, Netser S (2009). Patient characteristics and cell source determine the number of isolated human cardiac progenitor cells. Circulation.

[120] Cai B, Ma W, Bi C, Yang F, Zhang L, Han Z (2016). Long noncoding RNA H19 mediates melatonin inhibition of premature senescence of c-kit(+) cardiac progenitor cells by promoting miR-675. J Pineal Res.

[121] Saheera S, Potnuri AG, Nair R (2017). Histamine-2 receptor antagonist famotidine modulates cardiac stem cell characteristics in hypertensive heart disease. PeerJ.

[122] Saheera S, Potnuri AG, Nair RR (2018). Modulation of cardiac stem cell characteristics by metoprolol in hypertensive heart disease. Hypertens Res.

[123] Cantz T, Martin U (2010). Induced pluripotent stem cells: characteristics and perspectives. Adv Biochem Eng Biotechnol.

[124] Giorgetti A, Montserrat N, Rodriguez-Piza I, Azqueta C, Veiga A, Izpisua Belmonte JC (2010). Generation of induced pluripotent stem cells from human cord blood cells with only two factors: Oct4 and Sox2. Nat Protoc.

[125] Szabo E, Rampalli S, Risueno RM, Schnerch A, Mitchell R, Fiebig-Comyn A (2010). Direct conversion of human fibroblasts to multilineage blood progenitors. Nature.

[126] Banito A, Rashid ST, Acosta JC, Li S, Pereira CF, Geti I (2009). Senescence impairs successful reprogramming to pluripotent stem cells. Genes Dev.

[127] Berniakovich I, Laricchia-Robbio L, Izpisua Belmonte JC (2012). N-acetylcysteine protects induced pluripotent stem cells from in vitro stress: impact on differentiation outcome. Int J Dev Biol.

[128] Esteban MA, Wang T, Qin B, Yang J, Qin D, Cai J (2010). Vitamin C enhances the generation of mouse and human induced pluripotent stem cells. Cell Stem Cell.

[129] Son MJ, Son MY, Seol B, Kim MJ, Yoo CH, Han MK (2013). Nicotinamide overcomes pluripotency deficits and reprogramming barriers. Stem Cells.

[130] Schwartz SM, Benditt EP (1976). Clustering of replicating cells in aortic endothelium. Proc Natl Acad Sci U S A.

[131] Yun SP, Lee MY, Ryu JM, Song CH, Han HJ (2009). Role of HIF-1alpha and VEGF in human mesenchymal stem cell proliferation by 17beta-estradiol: involvement of PKC, PI3K/Akt, and MAPKs. Am J Physiol Cell Physiol.

